# Assessment of Anticancer Properties of *Argemone mexicana* L. and Berberine: A Comparative Study

**DOI:** 10.3390/plants13101374

**Published:** 2024-05-15

**Authors:** Joel H. Elizondo-Luevano, Ramiro Quintanilla-Licea, Imelda N. Monroy-García, Miroslava Kačániová, Uziel Castillo-Velázquez, Aldo F. Bazaldúa-Rodríguez, Lourdes M. Garza-Vega, Ángel D. Torres-Hernández, Abelardo Chávez-Montes

**Affiliations:** 1 Department of Chemistry, Facultad de Ciencias Biológicas (FCB), Universidad Autónoma de Nuevo León (UANL), Ciudad Universitaria, San Nicolás de los Garza 66455, Nuevo León, Mexico; ramiro.quintanillalc@uanl.edu.mx (R.Q.-L.); aldo.bazalduarg@uanl.edu.mx (A.F.B.-R.); lourdes.garzava@uanl.edu.mx (L.M.G.-V.); 2 Department of Chemical and Biochemical Engineering, Instituto Tecnológico de Los Mochis, Tecnológico Nacional de México (ITLM—TecNM), Juan de Dios Bátiz y 20 de Noviembre, Los Mochis 81259, Sinaloa, Mexico; imelda.mg@mochis.tecnm.mx; 3 Institute of Horticulture, Faculty of Horticulture and Landscape Engineering, Slovak University of Agriculture, Tr. A. Hlinku 2, 94976 Nitra, Slovakia; miroslava.kacaniova@gmail.com; 4 School of Medical & Health Sciences, University of Economics and Human Sciences in Warsaw, Okopowa 59, 01 043 Warszawa, Poland; 5 Department of Immunology, Facultad de Medicina Veterinaria y Zootecnia, UANL, Ex Hacienda del Cañada, Cd. General Escobedo C.P. 66054, Nuevo León, Mexico; uziel.castillovl@uanl.edu.mx; 6 Department of Microbiology and Immunology, FCB, UANL, Ciudad Universitaria, San Nicolás de los Garza 66455, Nuevo León, Mexico; angel.torreshr@uanl.edu.mx

**Keywords:** alkaloids, *Argemone*, *Artemia salina*, berberine, cytotoxicity, hemolysis, extracts, Mexican poppy, nitric oxide, Papaveraceae

## Abstract

*Argemone mexicana* L. has been used in traditional Mexican medicine. Among its bioactive constituents, berberine (BER) has garnered attention for its cytotoxic properties against different tumor cell lines. This study investigates the in vitro toxicity against HEP-G2 (human hepatocellular carcinoma) and murine lymphoma (L5178Y-R) cells using the MTT assay of the methanol extract (AmexM), sub-partitions of *A. mexicana*, and BER. Selectivity indices (SIs) were determined by comparing their cytotoxic effects on VERO (monkey kidney epithelial) and PBMC (human peripheral blood mononuclear) non-tumoral cells. Additionally, the anti-hemolytic effect of these treatments was assessed using the AAPH method. The treatment with the most promising activity against tumor cells and anti-hemolytic efficacy underwent further evaluation for toxicity in *Artemia salina* and antioxidant activities using DPPH, ABTS, and FRAP assays. BER demonstrated an IC_50_ = 56.86 µg/mL in HEP-G2 cells and IC_50_ < 5.0 µg/mL in L5178Y-R cells, with SI values of 15.97 and >5.40 in VERO and PBMC cells, respectively. No significant hemolytic effects were observed, although AmexM and BER exhibited the highest anti-hemolytic activity. BER also demonstrated superior antioxidant efficacy, with lower toxicity in *A. salina* nauplii compared to the control. Additionally, BER significantly attenuated nitric oxide production. This study highlights the antiproliferative effects of *A. mexicana*, particularly BER, against HEP-G2 and L5178Y-R tumor cell lines, along with its selectivity towards normal cells. Furthermore, its anti-hemolytic and antioxidant potentials were demonstrated, suggesting that BER is a promising candidate for potent chemotherapeutic agents.

## 1. Introduction

Plants possess extensive biological and medicinal properties, making them a valuable source of chemical compounds with potential therapeutic effects [1]. Moreover, plants are renowned for their high safety profile, wide availability, easy accessibility, and affordability [2,3]. Herbal medicine, an ancient practice across global cultures [4], incorporates both organic and inorganic materials not only from plants but also from animal and mineral sources [5]. This branch of traditional medicine encompasses a wide range of materials, including raw plant parts like leaves, flowers, and roots, as well as derived products such as juices, essential oils, and powders [6,7,8]. As a result, plants play a crucial role in providing a vast array of compounds that hold immense potential for various therapeutic applications [9,10].

According to the World Health Organization (WHO), 60% of the world’s population relies on herbal medicine, particularly in developing countries [11]. Phytochemicals and their analogs have yielded clinically useful drugs [12]. The herbal medicine industry generates USD 100 billion annually with a growth rate of 15% [11]. Despite its popularity, herbal medicine poses challenges in standardization and safety. As a result, clinical research efforts have intensified to validate its efficacy [13].

*Argemone mexicana* L. (Papaveraceae), commonly known as Mexican prickly poppy or chicalote, is a plant native to Mexico that has spread to tropical and subtropical regions worldwide [14]. It is revered for its medicinal properties, which include antimicrobial, antiparasitic, cytotoxic, and neurological effects [15]. These therapeutic properties are attributed to the presence of various benzylisoquinoline alkaloids, such as protoberberines like berberine (BER) and protopines [16,17]. Several studies have investigated the cytotoxic effects of isolated alkaloids from *A. mexicana* against various cancer cell lines, including human nasopharyngeal carcinoma (HONE-1), human gastric cancer (NUGC) [18], human lung epithelial (A-549), human colon adenocarcinoma (HT-29), and human promyelocytic leukemia (HL-60) cell lines [19].

Despite the numerous pharmacological studies conducted so far on many Papaveraceae species, not all species have been analyzed. In our workgroup, we have extensive experience evaluating Mexican plants, such as *A. mexicana*, among others [18,19,20]. We have previously published studies conducted with *A. mexicana* against different etiological agents, such as parasites, from which we identified and reported the benzylisoquinoline alkaloid BER as the main component of *A. mexicana* and which has antiparasitic and anthelmintic activity [20,21].

Berberine (BER) is a phytochemical present in medicinal herbs like *Berberis aristata*, *Berberis vulgaris*, *Coptis chinensis*, *Rhizoma coptidis*, and *A. mexicana* [22,23,24]. This isoquinoline alkaloid has numerous biological and pharmacological effects, including antioxidant, anti-inflammatory, antimicrobial, anthelmintic, hepatoprotective, hypoglycemic, and antiparasitic effects, among others [21,22]. Notably, various studies have suggested that BER could be a promising drug candidate with a wide range of therapeutic applications, such as antitumor and carcinogenicity [24]. Over the past few years, there have been reports on the ability of BER to hinder the growth of tumor cells, exhibit cytotoxic effects, and induce apoptosis in cancer cells [25]. This evidence has prompted numerous studies aimed at enhancing the effectiveness and selectivity of BER; the results obtained thus far from experiments conducted on human cancer cell lines suggest that BER holds promise as a potential treatment for cancer [26,27].

As a continuation of our previous studies based on the chemical and biological characterization of plant-derived extracts for potential use as phytotherapy [21,28]. In previous work, we discussed the in vitro cytotoxic activity of the crude methanolic extract of *A. mexicana* against HEP-G2 (human hepatocellular carcinoma) and L5178Y-R (murine lymphoma) cell viability [29,30]. Therefore, the main objective of the present investigation is focused on the report of the antiproliferative activity against some normal and cancer cell lines and anti-hemolytic properties of the methanolic extract of *A. mexicana*, fractions obtained from solvents of ascending polarity, and the alkaloid BER previously identified as the main component of *A. mexicana*. The lethality of *Artemia salina* nauplii, the antioxidant effect, and the nitric oxide (NO) production of the most effective treatments were also determined.

## 2. Materials and Methods

### 2.1. Chemicals and Reagents

Dulbecco’s modified eagle medium (DMEM culture medium), 1% *v/v* antibiotic/antimycotic solution, fetal bovine serum (FBS), and sodium bicarbonate (NaHCO_3_) were purchased from Gibco™ (Thermo Fisher Scientific Inc. Waltham, MA, USA). 2,2′-azino-bis(3-ethylbenzothiazoline-6-sulfonic acid) (ABTS), 2,2′-azobis (2-methylpropionamidine) dihydrochloride (AAPH), 2,2-diphenyl-1-picrylhydrazyl (DPPH), 3-(4,5-dimethylthiazol-2-yl)-2,5-diphenyltetrazolium bromide (MTT), 4-2(2-hydroxyethyl)-1-piperazine ethane sulfonic acid (HEPES), ascorbic acid (vitamin C), berberine chloride form (berberine, CAS: 633-65-8), chloroform-d (CDCl_3_), potassium persulfate (K_2_S_2_O_8_), ferric chloride, Griess reagent, L-glutamine, lipopolysaccharide (LPS) from *Escherichia coli* O26:B6, Roswell Park Memorial Institute medium (RPMI-1640 culture medium), sodium acetate acid, sodium bicarbonate (NaHCO_3_), sodium chloride (NaCl), sodium hydroxide (NaOH), sodium phosphate dibasic (Na_2_HPO_4_), sodium phosphate monobasic (NaH_2_-PO_4_), tripyridyltriazine (TPTZ), Tetramethylsilane (TMS), and vincristine sulfate (VS) salt (CAS: 2068-78-2) were obtained from Sigma-Aldrich^®^ (Merck KGaA, Darmstadt, DE, Germany). Brine shrimp eggs of *Artemia salina* Leach were purchased in INVE, Aquaculture INC (Salt Lake City, UT, USA). Chloroform (CHCl_3_), deuterated methanol (MeOH-d_4_), dimethyl sulfoxide (DMSO), iron (III) chloride hexahydrate, iron (II) sulfate heptahydrate, potassium dichromate (K_2_Cr_2_O_7_), hydrochloric acid (HCl), *n*-Hexane, and methyl alcohol (MeOH) were purchased from CTR^®^ Scientific (Control Técnico y Representaciones, SA de CV, NL, Mexico).

### 2.2. Cell Lines

Human hepatocellular carcinoma cells (HEP-G2; ATCC HB-8065 ™), murine lymphoma cells (L5178Y-R; ATCC CRL-1722 ™), non-tumoral monkey kidney epithelial cells (VERO; ATCC CCL-81 ™), and murine macrophage (J774A.1; ATCC TIB-67 ™) cells were obtained from the American Type Culture Collection (ATCC^®^, Manassas, VA, USA). Human peripheral blood mononuclear cells (PBMC) and human red cells (erythrocytes) were kindly provided by the Facultad de Medicina of the Universidad Autónoma de Nuevo León (UANL).

#### Ethics

The procedures employed in this study were approved by the UANL Ethics Committee, registration no. CI-01-22-2023 (Supplementary Materials). in compliance with the Official Mexican Technical Standard (NOM-253-SSA1-2012) [31]. The informed consent for healthy donors is also provided in the Supplementary Materials.

### 2.3. Plant Material and Extraction

This study presents findings on the cytotoxic activity of the methanolic extract of *A. mexicana* (AmexM) and sub-partitions of *n*-Hexane, CHCl_3_, and MeOH. The plant used in this study was identified with voucher number FCB-UNL 029128, which was previously identified and reported by us in doi: 10.3347/kjp.2020.58.2.135; a specimen was deposited at the herbarium of Facultad de Ciencias Biológicas (FCB), UANL. *A. mexicana* was collected in the city of Guadalupe, Nuevo León, Mexico, 25°39′40.6” N 100°11′02.0” W. The taxonomy of *A. mexicana* has been validated on the ThePlantList (TPL) and on the World Flora Online (WFO) websites (http://www.theplantlist.org; www.worldfloraonline.org; accessed on 11 May 2024).

#### 2.3.1. Extraction

An amount of 100 g of milled dry material was treated with 1000 mL of MeOH in a Soxhlet apparatus for 72 h [32] to produce a crude methanol extract (AmexM) [33]. The resulting soluble partitions were then obtained using the same equipment for 72 h, with solvents of increasing polarity used to produce *n*-Hexane (AmexHP), CHCl_3_ (AmexCP), absolute MeOH (AmexMP), and aqueous (distilled H_2_O, AmexAq) partitions [34]. The extracts and partitions were filtered (Whatman™ qualitative filter paper, grade 1; Cytiva, Global Life Sciences Solutions USA LLC, Marlborough, MA, USA). The extract (AmexM) and organic partitions (AmexHP, AmexCP, and AmexMP) were rotaevaporated in a RE200 rotary evaporator (Yamato Scientific Co., Ltd. Harumi, Chuo-ku, Tokyo, Japan) at 80 rpm and 40 °C in a water bath and stored at 4 °C in amber bottles until use [10]. On the other hand, the AmexAq partition was lyophilized (Free Zone 2.5 Liter -50C Benchtop Freeze Dryer, Labconco Corporation, Kansas City, MO, USA), and then dried and stored (protected from light in amber bottles) at −20 °C. The extraction yield percentages were calculated using Formula (1) as follows:

(1) $$Yield\;\%=\frac{Final\;weight}{Initial\;weight}\times 100$$

#### 2.3.2. Phytochemical Analysis

**Figure 1:**
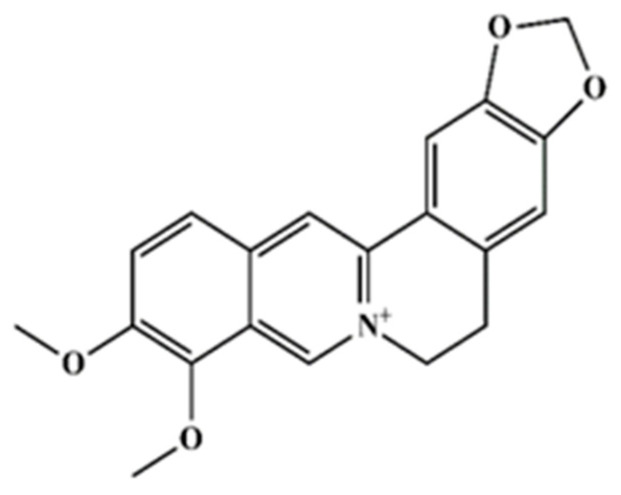
Structure of BER compound. Molecular formula: C_20_H_18_NO_4_^+^; molecular weight: 336.3612 g/mol; IUPAC name: 16,17-dimethoxy-5,7-dioxa-13-azoniapentacyclo [11.8.0.0^2,10^.0^4,8^.0^15,20^]henicosa-1(13),2,4(8),9,14,16,18,20-octaene. PubChem CID: 2353.

We previously reported BER (Figure 1) as the main component of *A. mexicana* in doi: 10.3347/kjp.2020.58.2.135, where the phytochemical tests of the extract of *A. mexicana* and the identification of BER were based on spectroscopic/spectrometric analysis and comparison with bibliographic data. BER structure was matched on the PubChem website (https://pubchem.ncbi.nlm.nih.gov/compound/2353; accessed on 11 May 2024). In the present study, we used the standard grade reagent BER (berberine chloride form, CAS: 633-65-8, Sigma-Aldrich^®^) for the subsequent biological analyses and assays.

The methodologies and tests performed for the corresponding phytochemical analysis were determined via high-performance liquid chromatography coupled with mass spectrometry (HPLC-MS) and nuclear magnetic resonance spectroscopy (NMR). The analyses were conducted as described in the following paragraphs.

##### A. HPLC-MS

The AmexM crude extract and standard analytical grade BER were subjected to analysis and comparison using high-performance liquid chromatography with a diode array detector (HPLC-DAD). In summary, 10 mg of the AmexM was dissolved in a 1 mL MeOH mixture and then filtered through a Millex^®^ 0.2 mm pore size nylon membrane (Merck Millipore^®^, Burlington, MA, USA). For the analysis, a Waters Alliance 2695 HPLC Separations Module (Conquer Scientific LLC., Poway, CA, USA) equipped with an in-line degasser, quaternary pump, autosampler, column temperature control module, and diode array detector was utilized. Separation was conducted on a Kinetex F5 (PFP 50 × 2.1 mm) column (Phenomenex Inc., Torrance, CA, USA) with a mobile phase consisting of an aqueous solution of formic acid (1%) and methanol. The gradient program commenced with 30% MeOH, maintained for 2 min, followed by a linear increase to 100% over 5 min. This concentration was held for one minute before returning to the initial conditions over two minutes. The reconditioning time between analyses was 10 min. The mobile phase flow rate was set at 400 μL/min, the column temperature was maintained at 50 °C, and the injection volume was 0.5 μL. To confirm the identities of the components identified in the active fractions, mass spectrometry analysis was performed via direct infusion using an LCQ Fleet (Thermo Fisher Scientific Inc., Stoughton, MA, USA) mass spectrometer equipped with an electrospray ionization source and an ion trap analyzer. Nitrogen served as the sheath gas at a flow rate of 30 units in the ionization source. Operating in positive mode, the voltage of the electrospray capillary was set to 5 kV, and the voltage of the desolvation capillary was set to 43 V at 275 °C. The lens tube voltage was maintained at 75 V. Data acquisition was conducted in full-scan mode across a mass-to-charge ratio (*m/z*) range of 100 to 1000. For the most intense ions, collision-induced dissociation (CID) mode was employed in mass/mass experiments, with the normalized collision energy adjusted to achieve adequate fragmentation using an insulation width of 1 *m*/*z*, an activation Q of 0.3, and an activation time of 30 ms [35].

##### B. NMR

^1^H-NMR and ^13^C-NMR were conducted using a Bruker Avance III™ HD 400 MHz Prodigy spectrometer (Bruker Corporation, Billerica, MA, USA) equipped with gradients and a 5 mm multinuclear probe. For analysis, BER and dried AmexM raw extract were dissolved in MeOH-d_4_ with TMS (0.3%) as a zero reference [36]. NMR spectra were analyzed using Topspin 3.0 software (Bruker Corp.). ^1^H-NMR spectra were recorded in CDCl_3_ and ^13^C-NMR spectra were recorded in MeOH-d_4_ [37].

### 2.4. Cell Viability Assays

The HEP-G2 cancer cell line and the normal VERO cell line were cultured in DMEM supplemented with 10% FBS, 2% NaHCO_3_, and HEPES. All tests performed with these cells were carried out in 96-well flat-bottom plastic microplates (Corning^®^ Labware and Equipment, Oneonta, NY, USA) due to the adherent nature of these cells [30]. L5178Y-R cells and PBMC were maintained in RPMI-1640 culture medium supplemented with 10% FBS and 1% antibiotic/antimycotic solution. All the tests performed with these cells were carried out in 96-well curved-bottom plastic microplates (Corning^®^) because these cells are non-adherent [29].

Prior to the application of the treatments, the cells were incubated at 37 °C in a humidified incubator (Sanyo MCO-19AIC CO_2_ Incubator, Sanyo Electric Co., Ltd., Gunma-ken, Japan) with 5% CO_2_ for 24 h for adaptation [38]. The cell viability was determined by MTT assay; MTT color intensity was directly associated with the number of living cells [39] after 72 h of incubation. Mitochondrial enzymes, specifically succinate dehydrogenase, reduce MTT tetrazolium salt to form formazan; this reaction produces a purple–blue product that can be measured using spectrophotometry since the in vitro cell viability can be tested using the MTT colorimetric assay [40]. Therefore, we decided to perform an MTT assay to correlate mitochondrial activity with viability. To test cytotoxicity, the cells were treated with concentrations of each treatment ranging from 31.25 µg/mL to 1000 µg/mL in a final volume of 200 µL for 48 h. The positive control consisted of 0.05 µg/mL on VS; the negative control was culture medium alone [41]. All treatments were diluted in DMSO to a final well test concentration not exceeding 0.2% (*v*/*v*) [42].

The mean inhibitory concentration (IC_50_) values were determined after 72 h of treatment incubations with the cells by measuring the absorbance (Abs) at 570 nm on a microplate reader (Thermo Fisher Scientific Inc., Stoughton, MA, USA). The selectivity indexes (SI) were obtained after dividing the IC_50_ of the normal cell on the IC_50_ of the respective tumor cell. Any sample with an SI value greater than 3 was considered high [38]. Cell viability and SI were determined by the following formulas, respectively (2) and (3):

(2) $$Cell\;viability\;\%=\frac{\;{Abs}_{570nm}Treatment}{\;{Abs}_{570nm}\;Negative\;control}\times 100$$

(3) $$SI=\frac{{IC}_{50}\;Normal\;Cells\;Value}{{IC}_{50}\;Tumor\;Cells\;Value}$$

### 2.5. Hemolytic and Anti-Hemolytic Activity

#### 2.5.1. Hemolytic Test

The hemolytic activity was assessed using the hemolysis test [43]. Treatments evaluated were prepared in PBS (pH 7.2 ± 0.2) in concentrations ranging from 10, 100, 200, 400, 600, 800, 1000, and 2000 µg/mL; the percent (%) of hemolysis was determined by measuring the Abs at 540 nm for each treatment. IC_50_ values were defined as the sample concentration needed to cause 50% hemolysis of human red blood cells and were computed using Formula (4):

(4) $$Hemolysis\;\%=\frac{{Abs}_{540nm}\;Treatment}{{Abs}_{540nm}\;Positive\;control}\times 100$$

#### 2.5.2. Anti-Hemolytic Test by the AAPH Assay

The AAPH inhibition test, as previously reported [44], was used to determine the anti-hemolytic activity. Hemolysis was induced by the AAPH radical (150 mM, prepared in PBS) as a positive control. The concentrations of the treatments were the same as in the hemolysis assay plus the AAPH. The IC_50_ values were defined as the sample concentration needed to cause 50% hemolysis and were calculated as follows (5):

(5) $$Anti-hemolytic\;Activity\;\%=100-\left(\frac{{Abs}_{570nm}\;Treatment}{{Abs}_{570nm}\;Positive\;control}\times 100\;\right)\;$$

### 2.6. Lethality in Artemia salina

The most effective treatments in the cytotoxicity test against tumor cells were tested for lethality in *A. salina* (brine shrimp) as an in vivo model assay, which was determined using the methodology described by Pérez-Hernández et al. in 2015 [45]. Artificial seawater was prepared using 20 g of sea salt and 6 mg of brewer’s yeast dissolved in 500 mL of distilled H_2_O (pH 7.8). Prior to the assay, the artificial seawater was conditioned by supplying air with an aquarium pump for 24 h. For the hatching of *A. salina* nauplii, a rectangular glass container (17 × 14 × 7 cm) was adapted, with a dark section where the cysts were incubated and an illuminated area that allows only hatched nauplii to be obtained by means of phototropism. After an incubation period of 48 h under room temperature conditions of 25 ± 2.0 °C (aeration and constant light), the test was carried out using 96-well transparent plastic microplates with a concave bottom (Corning^®^), in which 20 nauplii and different concentrations of the treatments (10, 100, 200, 400, 600, 800, 1000, and 2000 µg/mL) were deposited in a final volume of 200 µL per well [46]. After 24 h of exposure, the count of live and dead *A. salina* nauplii was recorded to determine the IC_50_ values. Counting of live and dead larvae in each well of the microplate was performed with the use of a stereoscope microscope. K_2_Cr_2_O_7_ at 100 µg/mL and artificial seawater were used as positive and negative controls, respectively. *A. salina* nauplii viability was determined by Formula (6) as follows:

(6) $$A.\;salina\;Viability\;\%=\frac{\;Survival\;Treatment\;Count}{\;Survival\;Control\;Count}\times 100$$

### 2.7. Antioxidant Activities

The antioxidant activity was determined by the DPPH, ABTS radical scavenging [38], and FRAP (Ferric Reducing Antioxidant Power) [47] methods. In the DPPH and ABTS assays, Vitamin C served as the positive control. In all treatment evaluations, the concentrations ranged from 15.63, 31.25, 62.50, 150, 250, 500, and 1000 µg/mL.

#### 2.7.1. DPPH Scavenging Test

The antioxidant activity was assessed using the DPPH radical assay [48], where the antioxidant activity (free radical scavenging capacity) was quantified as IC_50_ in µg/mL. IC_50_ represents the concentration of the test material required to cause a 50% decrease in the initial concentration of DPPH. The DPPH radical scavenging assay was conducted in a 96-well flat-bottom plastic microplate (Corning^®^). The percentage inhibition of DPPH at 517 nm was determined using a UV/VIS spectrophotometer and calculated using Formula (7) as follows:

(7) $$DPPH\;scavenging\;\%=\frac{{Abs}_{517}\;Control-{Abs}_{517}\;Sample}{{Abs}_{517}\;Control}\times 100\;$$

#### 2.7.2. ABTS Scavenging Test

The antioxidant activity was determined using the ABTS radical scavenging method [38], where the antioxidant activity (free radical scavenging capacity) was quantified as IC_50_ in µg/mL. IC_50_ represents the concentration of the test material required to cause a 50% decrease in the initial concentration of the ABTS radical. The ABTS radical scavenging assay was conducted in 96-well plastic microplates (Corning^®^), and the percentage inhibition of ABTS at 734 nm was calculated using Formula (8) as follows:

(8) $$ABTS\;scavenging\;\%=\frac{{Abs}_{734nm}\;Control-{Abs}_{734}\;Sample}{{Abs}_{734nm}\;Control}\times 100$$

#### 2.7.3. FRAP Scavenging Test

The FRAP assay, utilized to assess the antioxidant potential of compounds or natural extracts, relies on the ability of antioxidative compounds to reduce TPTZ-Fe^3+^ under acidic conditions, forming the stable ferrous form (TPTZ-Fe^2+^), which exhibits maximum absorbance at 593 nm. The assay was conducted following the methodology outlined by Huong-Huynh et al. in 2024 [49]. Fresh FRAP reagent was prepared by mixing 2.5 mL of a solution containing 10 mM TPTZ in 40 mM HCl with 2.5 mL of FeCl_3_.6H_2_O (20 mM) and 25 mL of acetate buffer (300 mM, pH 3.6). Subsequently, 40 μL of the treatment at varying concentrations and 1850 μL of FRAP reagent were combined, and the absorbance of the reaction mixture was measured at 593 nm. After a 30 min incubation period in the dark, the absorbance was measured again. MeOH was used as the reaction blank. The FRAP values were obtained using a standard calibration curve (percentage of Fe^3+^ scavenging reduction to Fe^2+^) using different FeSO_4_ (1.0 mM) solution concentrations. FRAP values are expressed as µmol Fe^2+^/mL concentrations.

### 2.8. Nitric Oxide Production

The nitric oxide (NO) assay was conducted on murine macrophages (ATCC TIB-67 ™, J774A.1 cell line) [50], which were cultured for 24 h with concentrations ranging from 0.00, 0.98, 1.95, 3.91, 7.81, 15.63, 31.25, 62.50, 150, 250, 500, and 1000 µg/mL of the most effective treatment against tumor cells and anti-hemolytic activity. The macrophage cultures were incubated in triplicate in 25 cm^2^ tissue culture flasks (Corning Glass Works, Corning^®^, Oneonta, NY, USA) in a total volume of 7 mL of RPMI-1640 culture medium supplemented with 10% FBS and 1% antibiotic/antimycotic solution and maintained at 37 °C in 5% CO_2_. A concentration of 200 ng/mL of *E. coli* O26:B6 LPS served as an inflammatory-inducing agent to stimulate NO production. NO production was assessed by measuring nitrite accumulation in the supernatant using Griess reagent. A standard curve was generated using NaNO2 (1 M) to interpret the test results.

### 2.9. Statistical Analysis

Data are shown as the mean ± SD. A 1-way ANOVA test was employed to determine the significant differences. Tukey’s or Dunnett’s post hoc tests were used when required. The IC_50_ and LD_50_ values were calculated by the Probit test. All assays were conducted in triplicate at least three times. We used the Statistical Package for the Social Sciences (SPSS) software, version 24.0 (IBM Inc. Armonk, NY, USA), for statistical analyses.

## 3. Results

### 3.1. Phytochemical Data of Argemone mexicana

**Figure 2:**
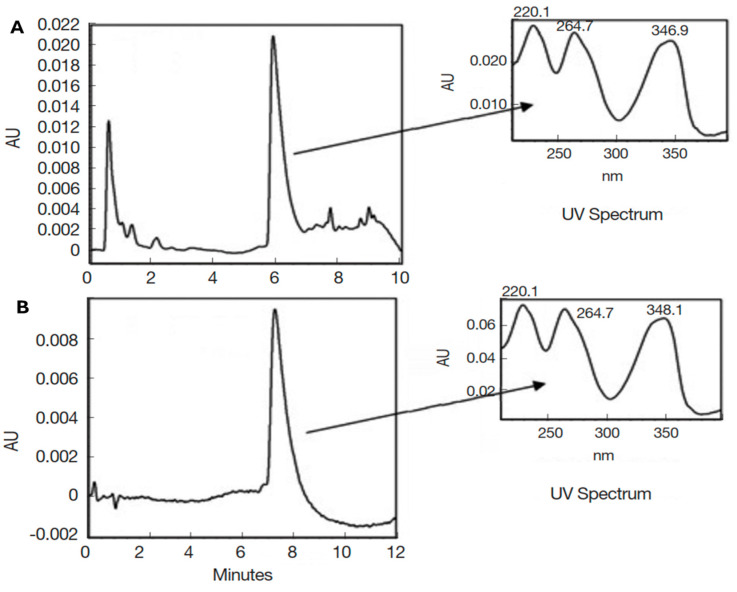
HPLC-DAD chromatograms for (**A**) AmexM and (**B**) BER, standard grade (*m/z* 336.36). The Y-axis shows the absorbance units (AUs).

**Figure 3:**
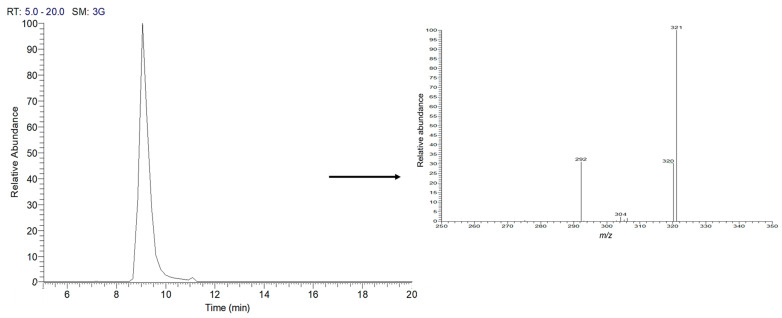
Direct-infusion electrospray ionization ion trap MS^2^ full scan product ion mass spectra of M^+^ ions of BER (chromatograms and mass spectra *m*/*z* 336.36) obtained from the AmexH extract. Retention times are shown in min. The Y-axis shows the relative abundance.

As indicated in the methodology section, in this study, the crude methanol extract of *A. mexicana* (AmexM), as well as its partitions obtained with solvents of increasing polarity, were evaluated to provide a broad approach to the biological activity of this plant since, with different plants, certain advantages have been observed when partitioning the extract with solvents of different polarities and evaluating them in in vitro biological studies [51,52]. Table 1 shows the percent (%) of extraction yield of the extract and sub-partitions. Our research group previously reported the identification of BER as the main secondary metabolite of *A. mexicana* [35,36]. The identification of BER was based on spectroscopic/spectrometric analysis and comparison with bibliographic data. Figure 2 and Figure 3 show the liquid chromatography–mass spectrometry analyses of AmexM and BER (standard grade), which were analyzed and compared by HPLC-DAD.

#### H-NMR and ^13^C-NMR

**Figure 4:**
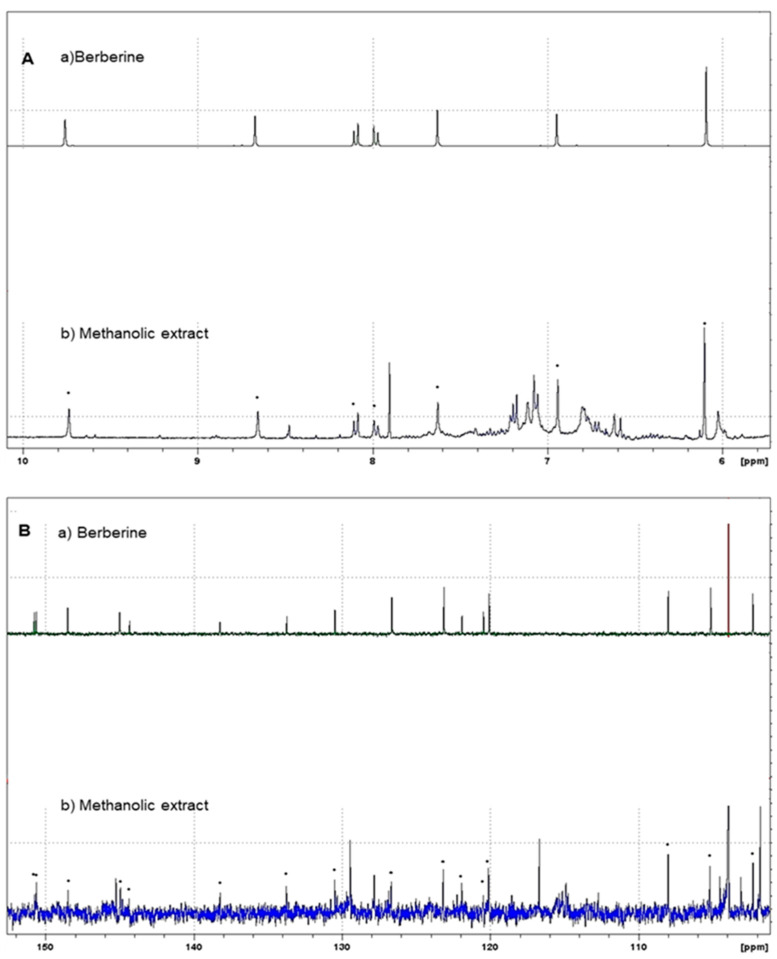
(**A**) ^1^H-NMR and (**B**) ^13^C-NMR spectra of BER (subfigures **Aa**,**Ba**) and AmexM (subfigures **Ab**,**Bb**). The (**•**) dots identify the signals corresponding to BER.

In this study, from the methanol extract of *A. mexicana*, the *n*-Hexane, CHCl_3_, MeOH, and H_2_O partitions were obtained. The composition of the main component of *A. mexicana*, the alkaloid berberine, was determined by spectrophotometric and NMR methods. Figure 4 shows the ^1^H and ^13^C NMR spectra. Determinations were carried out in a Bruker Avance III™ HD 400 MHz Prodigy spectrometer (Bruker Corp.).

### 3.2. Cytotoxic Activity and SI

Table 2 presents the cytotoxicity outcomes of the extracts on both tumor and healthy cells, along with the corresponding selectivity indices (SIs) for each extract. HEP-G2 cells were compared to VERO cells due to their adherence characteristics, while L5178Y-R cells were contrasted with PBMC cells as they are non-adherent. The AmexM extract was separated using *n*-Hexane extraction, which produced a residue (AmexHP); subsequently, the insoluble residue was dissolved in CHCl_3_ (AmexCP) and then the insoluble residue was dissolved in MeOH. For additional processing of the methanol residue (AmexMP), we obtained an aqueous fraction (AmexAP).

AmexM showed low effectiveness against HEP-G2 cells (IC_50_ 1020.77 µg/mL); however, against L5178Y-R, it showed good mean inhibitory activity (IC_50_ = 70.73 µg/mL). The AmexHP, AmexCP, AmexMP, and AmexAP subfractions showed no mean cytotoxic activity against PBMC (IC_50_ > 1100 µg/mL); however, against VERO cells, only AmexCP showed some activity with IC_50_ = 64.64 µg/mL. AmexMP and AmexAP treatments presented the lowest SI on HEP-G2 and L5178Y-R tumor cells (SI = 0.83 and 0.32, respectively).

AmexM presented a good SI and mean inhibitory activity against L5178Y-R cells (SI = 5.63, IC_50_ = 70.73 µg/mL) but not against HEP-G2 wings (SI = 0.49, IC_50_ = 1020.77 µg/mL). The highest SIs against HEP-G2 cells corresponded to BER, with SI of 15.97 showing an IC_50_ = 56.86 µg/mL on HEP-G2 cells and IC_50_ = 908.17 µg/mL on VERO cells. The highest SIs against L5178Y-R cells corresponded to AmexM and BER, which showed SIs of 5.63 (IC_50_ = 70.73 µg/mL) and > 5.40 (IC_50_ = 27.14 µg/mL), respectively.

### 3.3. Hemolytic and Anti-hemolytic Activity

For toxicity in erythrocytes, as well as for anti-hemolytic activity by protection against the radical AAPH in human erythrocytes (Table 3), the extract, fractions, and BER were tested. Regarding hemolytic activity, it was determined that the treatments showed no hemolytic effect on erythrocytes; the IC_50_ determined ranged from 712.74 µg/mL to 5309.10 µg/mL. For the anti-hemolytic activity assay, the treatments with the best cytoprotective effect were AmexM and BER with IC_50_ values of 32.85 and 36.88 µg/mL, respectively, and the treatment with the lowest effect was AmexCP with IC_50_ = 1359.79 µg/mL. Therefore, the AmexM and BER treatments were tested for antioxidant activity and lethality in *A. salina*.

### 3.4. Effect on A. salina and Antioxidant Activity

After determining the treatments’ effects on tumor cells, normal cells, and their toxicity in human erythrocytes, we evaluated the effects of AmexM and BER on lethality in *A. salina* nauplii and the antioxidant activities by the DPPH, ABTS, and FRAP methods (Table 4). Table 4 shows that the treatment with the best antioxidant activity was BER, with significantly higher activity (*p* < 0.05) compared to the positive control in the DPPH and ABTS tests. When lethality in *A. salina* nauplii was evaluated, both treatments were significantly (*p* < 0.001) better than the positive control; however, the AmexM treatment was significantly less toxic than BER (LD_50_ = 178.00, *p* < 0.05).

### 3.5. NO Production

**Figure 5:**
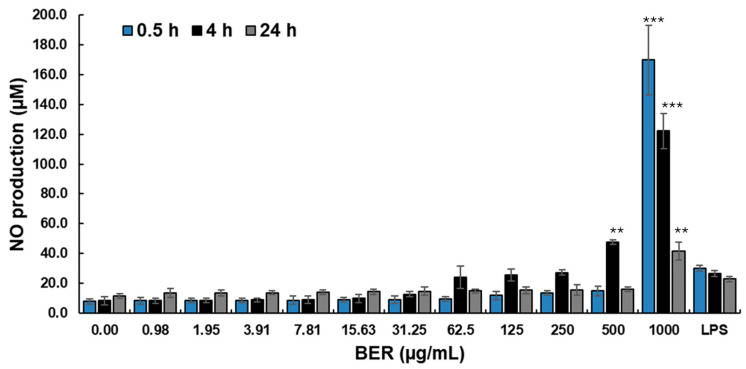
Nitric oxide (NO) production in µM caused by BER (berberine at 0.00–1000 µg/mL) and the *E. coli* LPS inflammatory inducer (200 µg/mL) determined at 0.5, 4, and 24 h. ** *p* < 0.01, *** *p* < 0.001 compared to their respective controls by Dunnett’s test.

In this investigation, we determined the effect on in vitro NO production evaluated in murine J774A.1 macrophages, which was provoked by the most effective treatment against tumor cells, AAPH assay, and antioxidant activity, which was BER. Figure 5 shows the effect on the macrophages in which the NO production capacity was evaluated using *E. coli* LPS (200 µg/mL, positive control) as an in vitro inflammation inducer. It can be observed that BER at 1000 µg/mL increased NO production in macrophages compared to the LPS inflammation control at 0.5, 4, and 24 h; at 0.5 h at concentrations of 0.00–500 µg/mL, no significant increase in NO production in macrophages was observed compared to LPS; at 4 h at concentrations of 0.00–31.25 µg/mL, there was no increase in NO production compared to LPS; at concentrations of 62.50–250 µg/mL, BER behaved in the same way as LPS; and only at 500–1000 µg/mL, was there an increase in NO production greater than that of LPS. However, at 24 h of incubation at concentrations of 0.00–500 µg/mL, there was no increase in ON production in macrophages compared to LPS.

## 4. Discussion

Contemporary medicine based on medicinal plants has become an area of growing interest internationally [11]. Mexico is known for its rich biodiversity, which includes a wide variety of plants with traditional medicinal properties. These plants have been used for centuries by indigenous and local communities to treat a wide range of ailments [53]. Some Mexican medicinal plants have demonstrated antioxidant, anti-inflammatory, antimicrobial, antiviral, analgesic, and anticancer properties, among others [12]. This has led to greater recognition and acceptance of medicinal plants in the medical field and among the general population. It represents a promising field that combines traditional knowledge with modern scientific research to improve health and well-being [54].

The analysis by HPLC is one of the most applied techniques to determine the compounds present in plants [55]. Characterization of BER, the main component of *A. mexicana*, can be easily identified using the HPLC technique [55]. The HPLC chromatograms of the analyzed AmexM are shown in Figure 2 and Figure 3; additionally, based on the NMR spectra, BER was also determined as the main component with at least 95% based on the ^1^H-NMR and ^13^C-NMR spectra comparing their spectroscopic data with those described previously in the literature (Figure 4) [36,56]. Quantification was performed through the standard calibration process using the reference standard compound berberine chloride [57]. The main identified compound in the analyzed sample (AmexM) turned out to be BER (Figure 2, Figure 3 and Figure 4), which has therapeutic uses [58] such as antioxidant [59], anti-inflammatory [60], antimicrobial, amebicidal/antihelminthic properties [44,61], as well as antineoplastic activity [56]. Similarly, methanol extracts and their partitions of *A. mexicana* have shown antimicrobial, antioxidant, antiparasitic [62], and cytotoxic potential [63].

In this study, we determined that all treatments, from the crude extract to the partitions, exhibited cytotoxic activity against the evaluated tumor cells HEP-G2 and L5178Y-R. However, berberine was the most effective treatment. Additionally, we calculated the selectivity index (SI) of these treatments in VERO and PBMC cells and found SIs of up to >15 for BER. This result suggests the promising selective effect of some treatments, as it has been indicated that SIs greater than 2 or 3 are promising [64,65].

In our study, the MTT (3-[4,5-dimethylthiazol-2-yl]-2,5-diphenyltetrazolium bromide tetrazolium) assay was used as it is widely employed in investigations to assess the cytotoxic activity of chemical and natural compounds due to its ability to provide an indirect measure of cell viability [65,66]. Although the MTT assay measures cellular metabolic activity rather than direct cytotoxicity, it can provide valuable information on the effect of a compound on the health and viability of cells [40,67].

Previous research has indicated that the 95% ethanolic extract of *A. mexicana* effectively hindered the proliferation of various cell lines, including A-549 (human pulmonary epithelial cell), HeLa-B75 (uterine cervix cell), HT-29 (human colon adenocarcinoma cell), HL-60 (human promyelocytic leukemia), and PN-15 (renal carcinoma), upon exposure to the extract [19]. Additionally, another study found that the aqueous extract of *A. mexicana* encapsulated in gold nanoparticles exhibited antiproliferative effects (IC_50_ = 12.03 μg/mL at 48 h) and genotoxic effects on human colon cancer cells (HCT-15) by suppressing cell growth and inducing apoptosis through the activation of p53 and caspase-3 genes [68].

Several derivatives of berberine have undergone evaluation against various human cancer cell lines, including prostate cancer (DU145 and PC3) and colon cancer (HT-29 and HCT-116), demonstrating significant antiproliferative effects with notable selectivity indices (>20). Furthermore, these compounds arrested the cell cycle at the G1 phase, markedly suppressed cell migration, and induced substantial cytoplasmic vacuolization [69]. This indicates a mechanism of action distinct from that of BER, which is known to bind to the molecular active site similarly to colchicine [70]. BER has been shown to inhibit the migration of HeLa cells, and its anticancer activity may, in part, stem from its ability to inhibit tubulin and microtubule assembly, underscoring its potential as an effective anticancer agent. Tubulin, the principal constituent of microtubules, is pivotal in cell division [71], and any disruption in its function results in mitotic arrest and cell cycle interruption [72].

Some studies have reported that natural compounds in plants as well as in extracts can be synergistically potentiated, which would indicate the cytotoxic effect of the crude extract as well as of the partitions (Table 2); moreover, investigations with isolated plant compounds have indicated that there is a synergistic effect between crude extract and its partitions [29]. For example, the alkaloid magnoflorin present in plants of the Papaveraceae and Berberidaceae family in combination with cisplatin increased its anticancer action and produced synergistic pharmacological interactions against cells of some types of breast, lung, rhabdomyosarcoma, and glioblastoma cancers [73].

Medicinal plants contain a plethora of bioactive compounds, including flavonoids, polyphenols, saponins, polysaccharides, triterpenoids, alkaloids, glycosides, and phenols. These compounds can synergistically inhibit tumor cell proliferation through various mechanisms, such as blocking cell cycle checkpoints and promoting apoptosis by activating caspases [74]. Additionally, they exhibit antioxidant, anti-inflammatory, and antiangiogenic effects. Moreover, natural substances have been found to effectively suppress early and intermediate stages of carcinogenesis and are generally well tolerated by cancer patients with minimal side effects [75].

A study in which extracts of different parts of *A. mexicana* were evaluated against a variety of tumor cells indicated that it had an effective cytotoxic effect against these cells similar to that of berberine [76]; this effect may be due to the combination of different components such as the benzylisoquinoline alkaloids BER, protopine, dihydrocoptisine, and jatrorrhizine. Therefore, this could indicate the effect of the crude extract of *A. mexicana* evaluated in the present investigation, as well as the effect of some of the sub-partitions against some of the cell lines.

Regarding the diversity of the cell lines used in our study, we are aware that the comparison between tumor and healthy cells from different species and tissues may raise questions about the consistency of the results. Ideally, the comparison between cell lines should be performed within the same species and tissue; however, this may be limited by the availability of biological material and resources available for the study. Our selection of cell lines was based on previous literature and, as a result, a comparison between adherent and non-adherent cells as well as SI was performed [30,41]. Our intention was to explore cellular properties related to cell adhesion in a broader context, as this feature may be relevant in cancer development and progression [64,77].

The hemolytic and anti-hemolytic determination test using the AAPH oxidative radical in vitro is a method used to evaluate the ability of certain substances to induce or prevent the lysis of red blood cells (erythrocytes) [41], as well as to assess the resistance of red blood cells to oxidation and the ability of certain substances to protect against oxidative stress-induced hemolysis [78]. These tests are used in biomedical and pharmacological research to understand the effect of compounds on the integrity of cell membranes caused by treatments such as plant extracts or natural products [79]. Table 3 presents the results corresponding to tests in human erythrocytes, where the chemoprotective effect of extracts, partitions, and BER was determined compared to the oxidative radical AAPH, which can cause damage to cell membranes and lead to red blood cell lysis [80]. The extract, fractions, and berberine were evaluated in erythrocytes in vitro, and it was found that the treatments did not present significant hemolytic activity. However, fractions obtained from AmexM were found to be less toxic in erythrocytes. When the effect against AAPH was determined, AmexM and berberine were found to be the most effective compared to the partitions. Our data are consistent with previous studies that investigated the antioxidant activities of the alkaloids berberine, jatrorrhizine, and magnoflorine isolated from *Mahonia aquifolium* using DPPH and AAPH tests, suggesting that these alkaloids may have potential as natural antioxidants [81]. Another study showed that BER protected neural stem cells (C17.2) from AAPH-induced damage and then promoted their differentiation into neurons, suggesting that berberine is a promising compound for the treatment of neurodegeneration [82].

Regarding antioxidant action, which was evaluated with the most effective treatments in cell toxicity and erythrocyte tests, this was determined by the ABTS, DPPH, and FRAP tests. The results are shown in Table 4, where it can be observed that BER was significantly more effective, even compared to controls. Determining antioxidant activity in vitro provides important information about the potential of different substances to combat oxidative stress, which may be relevant for the prevention and treatment of various diseases related to oxidative stress, such as cardiovascular diseases, neurodegenerative diseases, and cancer [83,84]. In this study, we investigated berberine hydrochloride’s in vitro antioxidant capacity. The results indicated that berberine has a potent in vitro antioxidant capacity, consistent with previous studies that evaluated berberine hydrochloride in vitro and demonstrated significant reducing capacity and radical scavenging effects, especially on ABTS (IC_50_ = 565.98 µg/mL) and DPPH (IC_50_ = 158.99 µg/mL) radicals, as well as by the FRAP method (IC_50_ = 751.82 µg/mL) [59]. Previous studies on neural stem cells C17.2 have shown that BER can protect cells from oxidative damage by reducing reactive oxygen species (ROS) levels and apoptotic factors such as Caspase 3, Bcl2, and Bax. Additionally, BER increases the expression of antiapoptotic factor Bcl2, which further reduces cell apoptosis. BER also promotes cell viability and differentiation and enhances the levels of pro-neural factors such as ASCL1, NeuroG1, NeuroD2, and DCX [82].

Regarding the toxicity model with *A. salina* described in this study, this has been widely used in toxicology to evaluate the risks of using various substances, including plant extracts, as it is an easy and economical technique that can also provide guidance on the toxicity of many natural compounds, drugs, and extracts [46,85]. A prior investigation assessing the MeOH extract of *Chelidonium majus* (*Papaveraceae*) revealed significant activity on *A. salina* larvae and colon carcinoma cells (HT-29), highlighting the concentrated cytotoxicity within the basic extract. The LD_50_ values were 250 µg/mL in *A. salina* and IC_50_ values of 1.14 µg/mL in HT-29 cell proliferation [86]. Furthermore, chromatographic separation of the ethanol extract on a large silica gel column yielded an active fraction, wherein the LD_50_ values for cytotoxicity were 98 µg/mL in *A. salina* and IC_50_ values of 0.49 µg/mL in the HT-29 cells. In this study, when comparing toxicity data obtained in normal cell cultures compared to *A. salina*, it is appreciated that the LD_50_ is higher for AmexM (LD_50_ = 570.65 µg/mL) compared to VERO (IC_50_ = 245.41 µg/mL) and PBMC (IC_50_ = 398.45 µg/mL). Although BER was significantly more antioxidant compared to AmexM, in the *A. salina* assay, BER was slightly more toxic (LD_50_ = 178.00 µg/mL) compared to AmexM (LD_50_ = 570.65 µg/mL, *p* < 0.05), so it is important to consider the toxicity effects on cells, as well as the SI.

The determination of nitric oxide (NO) in cellular assays is crucial in biomedical and medical research. NO is a reactive molecule that acts as a significant cellular messenger in a variety of physiological and pathological functions [87]. For the in vitro determination of NO, cells are cultured under specific conditions and exposed to stimuli that induce NO production [88]. For example, they may be treated with LPS or interleukin-1 (IL-1) [89]. The overproduction of NO causes tissue damage and is associated with chronic inflammation [90]. Some natural components present in herbal extracts have been shown to effectively inhibit LPS-induced NO in murine macrophages [38]. Therefore, as reported in Figure 5, BER exhibits immunomodulatory activity in response to NO production at concentrations of 0.98 to 500 µg/mL. However, at 1000 µg/mL it had a significantly greater effect on NO production compared to LPS. This could be due to BER inducing alternative macrophage activation [91]; further studies are needed to confirm this.

Based on the results presented, overall, this study provides valuable insights into the pharmacological properties of *A. mexicana* and BER, paving the way for future research and the development of new therapeutic agents for the treatment of cancer and related conditions. It is important to highlight the significance of considering the synergy of phytochemical compounds in extracts or partitions; the interaction between the various phytochemicals present in plants can have a significant impact on their biological activity. Moreover, the synergy between phytochemical compounds can potentially enhance their therapeutic effects, which is an important area of research in phytotherapy [29,92].

Therefore, considering the great future prospects of herbal medicaments, in the present investigation we reported the biological effect of Mexican poppy (*A. mexicana*) extracts, partitions, and BER according to their effects in different models both in vitro and in vivo. In addition, the need for future research in the development of herbal drugs as modern therapeutic agents is addressed.

## 5. Conclusions

The findings of this study demonstrate the cytotoxic effects of *A. mexicana* extracts, fractions, and BER on HEP-G2 and L5178Y-R cells. Particularly noteworthy is the potent cytotoxicity of BER, the primary compound found in *A. mexicana*, suggesting its potential as an antineoplastic agent. BER also exhibits remarkable anti-hemolytic and antioxidant properties, along with high selectivity rates compared to normal non-tumoral cells. Further investigations are warranted to elucidate the underlying mechanisms of action of *A. mexicana* and BER, as well as to evaluate their potential as natural sources of anticancer compounds.

According to our results, the compounds present in the AmexH extract present possible new methods of treatment of some pathologies such as neoplasms. However, it is important to verify our results by in vivo toxicity assays in higher organisms, as well as determine the associated molecular mechanisms. This study presents the first partial characterization of the extract of *A. mexicana.* The evaluation of the toxicity capacity in cells and erythrocytes of each fraction of the *A. mexicana* extract is presented with the results of the toxicity in *A. salina*.

## Figures and Tables

**Table 1:** Yield percentages of extraction.

Treatments	Abbreviation	Yield (%)
*A. mexicana* Ext. MeOH	AmexM	17.63
*A. mexicana* Fr. Hex	AmexHP	3.43
*A. mexicana* Fr. CHCl_3_	AmexCP	0.38
*A. mexicana* Fr. MeOH	AmexMP	1.86
*A. mexicana* H_2_O	AmexAq	11.96
Berberine	BER	^¶^

%: Yield percentage of extraction; ^¶^: not applicable since this was purchased as a standard grade reagent for biological activities.

**Table 2:** Cytotoxic activity by MTT assay and selectivity indices.

Treatments	IC_50_ (µg/mL) in Cells		SI	IC_50_ (µg/mL) in Cells		SI
	VERO	HEP-G2		PBMC	L5178 Y-R	
AmexM	245.41 ± 13.05 ^c^	1020.77 ± 21.74 ^d^	0.24 ^a^	398.45 ± 8.01 ^b^	70.73 ± 2.40 ^b^	5.63 ^c^
AmexHP	120.36 ± 2.66 ^b^	45.48 ± 8.07 ^b^	2.64 ^c^	>1200	155.21 ± 14.93 ^d^	>7.70 ^d^
AmexCP	64.64 ± 5.18 ^a^	17.96 ± 1.59 ^a^	3.59 ^d^	>1200	95.90 ± 3.19 ^c^	>10.00 ^e^
AmexMP	380.78 ± 12.91 ^d^	459.87 ± 6.39 ^c^	0.83 ^b^	>1200	573.83 ± 21.87 ^e^	>2.00 ^b^
AmexAP	550.07 ± 17.12 ^e^	1156.19 ± 18.62 ^e^	0.32 ^a^	1173.15 ± 74.90 ^c^	1094.06 ± 96.03 ^f^	1.07 ^a^
BER	908.17 ± 31.86 ^f^	56.86 ± 9.45 ^b^	15.97 ^e^	27.14 ± 7.16 ^a^	<5.0 ^a^	>5.40 ^c^
*p*—ANOVA	<0.01	<0.001	<0.001	<0.05	<0.001	<0.01

The mean IC_50_ values in µg/mL against the assessed cell lines are means ± SD, with significant differences (*p* < 0.05) indicated by different letters in the columns (Tukey’s test). SI values were obtained after 72 h of incubation, using 0.05 µg/mL vincristine sulfate (VS) as a positive control. IC_50_ values greater than 1200 µg/mL were not considered for Tukey’s analysis.

**Table 3:** Hemolytic and anti-hemolytic activities.

Treatment	Hemolytic Activity	Anti-Hemolytic Activity
	IC_50_ (µg/mL) in Erythrocytes	
AmexM	973.88 ± 38.46 ^b^	32.85 ± 11.21 ^a^
AmexHP	3479.80 ± 236.19 ^e^	79.93 ± 4.22 ^b^
AmexCP	2163.63 ± 214.76 ^c^	1359.79 ± 116.10 ^d^
AmexMP	5309.10 ± 131.17 ^f^	73.04 ± 10.33 ^b^
AmexAP	2924.24 ± 125.71 ^d^	259.01 ± 31.73 ^c^
BER	712.74 ± 37.98 ^a^	36.88 ± 5.49 ^a^
*p*—ANOVA	<0.001	<0.001

Data are mean ± SD of the IC_50_ values measured in µg/mL. Different letters in the columns indicate significant (*p* < 0.05) differences (Tukey’s test).

**Table 4:** Lethal activity on *A. salina* and antioxidant activity assays.

Treatments	*A. salina*	DPPH	ABTS	FRAP
	LD_50_ in µg/mL	IC_50_ in µg/mL	IC_50_ in µg/mL	IC_50_ in µmol Fe^2+^/mL
AmexM	570.65 ± 11.19 ^c,^***	565.98 ± 17.60 ^c^	158.99 ± 5.65 ^c^	751.82 ± 47.93 ^b^
BER	178.00 ± 29.70 ^b,^**	44.80 ± 1.22 ^a,^*	40.29 ± 9.02 ^a,^*	10.27 ± 2.04 ^a^
Vitamin C	−	68.90 ± 3.11 ^b^	81.76 ± 6.30 ^b^	−
K_2_Cr_2_O_7_	29.44 ± 4.61 ^a^	−	−	−
*p*—ANOVA	<0.001	<0.01	<0.01	<0.001

Data are mean ± SD of the LD_50_ or IC_50_ values. Different letters within the same column indicate significant (*p* < 0.05) differences (Tukey’s test). Positive control: Vitamin C in the DPPH and ABTS assays; K_2_Cr_2_O_7_ in the *A. salina* test. * *p* < 0.05, ** *p* < 0.01, *** *p* < 0.001 compared to their respective controls indicated in the methodology section and determined by the Dunnett’s test.

## Data Availability

The data availability statement is available from the corresponding author.

## Supplementary Materials

The following supporting information can be downloaded at: https://www.mdpi.com/article/10.3390/plants13101374/s1, Institutional Board Approval and Informed Consent Statement.

## Abbreviations

%: Percent; °C: Celsius degrees; µg/mL: micrograms per milliliter; µM: micromole; AAPH: 2,2′-azobis (2-methylpropionamidine) dihydrochloride; AmexAq: *A. mexicana* H_2_O fraction; AmexCP: *A. mexicana* CHCl_3_ fraction; AmexHP: *A. mexicana n*-Hexane fraction; AmexM: *A. mexicana* crude MeOH extract; AmexMP: *A. mexicana* MeOH fraction; ANOVA: analysis of variance; ATCC: American Type Culture Collection; AUs: absorbance units; BER: berberine; CHCl_3_: chloroform; CO_2_: carbon dioxide; DMEM: Dulbecco’s modified eagle medium; DPPH: 2,2-diphenyl-1-picrylhydrazyl; EtOAc: ethyl acetate; Ext: extract; FBS: fetal bovine serum; HEP: human hepatocarcinoma cells; HPLC: high-performance liquid chromatography; IC_50_: half maximal inhibitory concentration; LD_50_: half maximal lethal concentration; LPS: lipopolysaccharide; MeOH: methyl alcohol; MTT: 3-(4,5-dimethylthiazol-2-yl)-2,5-diphenyltetrazolium bromide; NMR: nuclear magnetic resonance spectroscopy; NO: nitric oxide; PBMC: human peripheral blood mononuclear cells; SD: standard deviation; SI: selectivity indices; UV: ultraviolet; VERO: African green monkey kidney cells.
