# Supplementary material for: Assessment of Anticancer Properties of Argemone mexicana L. and Berberine: A Comparative Study
Source: Plants (Basel). 2024 May 15;13(10):1374. doi: 10.3390/plants13101374 (PMC11125357; doi:10.3390/plants13101374)
Supplement: Supplementary file 1 [file plants-13-01374-s001.zip › Institutional Board Approval.pdf]

SUBJECT: Research project evaluation

Dr. Joel H. Elizondo Luevano

By this means, you are informed that in a session held on january 22nd, 2023, the research project with registration number CI-01-22-2023 "Antitumor activity of methanolic extracts of Mexican plants and soil and endophytic microorganisms on tumor cell lines" was reviewed and approved.

The global scientific evaluation of this research project complies with technical-scientific quality, scientific relevance and impact, as well as with the criteria, guidelines and institutional requirements.

Sincerely yours,

Yours
"*ALERE FLAMMAM VERITATIS*"
Cd. Universitaria
January 22nd, 2023

Research Committee

Dr. Juan Francisco Contreras
President

Dra. María Julissa Ek Ramos
Secretary
